# Association between initial microcirculation disturbance patients and mortality in patients who are critically ill: A retrospective cohort study

**DOI:** 10.1097/MD.0000000000035500

**Published:** 2023-10-27

**Authors:** Tongwu Guo, Rui Zheng, Huanying Yi, Yuanzheng Yang

**Affiliations:** a Emergency and Trauma College, Hainan Medical University, Haikou, China; b Brief introduction of International Nursing School, Hainan Medical University, Haikou, China; c The First Affiliated Hospital of Hainan Medical University, Haikou, China.

**Keywords:** anaerobic metabolism, arterial-venous oxygen content difference, lactic max level, mortality, tissue hypoxia, venous-arterial carbon dioxide difference

## Abstract

Impact of microcirculation status from mortality of critically ill population has been investigated for decades, but the prognosis of early initial microcirculation disturbance in critically ill population in the intensive care unit remains to be explored. The cohort study was conducted using the medical information database for intensive care IV. Critically ill adult in intensive care unit have been enrolled and categorized by early microcirculation status. Cox Proportional-Hazards models have been utilized for testing intermediaries and assess the relationship between combined early initial microcirculation disturbance and mortality. Several 2286 patients were initially screened. Some patients with a highest lactate level >2.2 mmol/L on the firstly day of admission (n = 1468) were then extracted for further analysis. 735 patients received in the initial microcirculation disturbance group as well as 733 patients were in the hyperlactatemia group. In those with elevated lactate, the 28-day mortality of early microcirculation disturbance was higher than that of hyperlactatemia alone (7-day mortality [16.19% vs 12.68%; Adjusted hazard ratio 1.35, 95% confidence intervals 1.03 to 1.78, *P* = .029], 28-day mortality [33.33% vs 27.28%; adjusted HR 1.34, 95% confidence interval 1.11 to 1.67, *P* = .002]). Early microcirculatory disturbances (increased P_V-A_CO_2_/C_A-V_O_2_ ratio and higher initial blood lactate level) were more reliable predictors of in-hospital mortality than early isolated lactate elevation.

## 1. Introduction

Patients with critical illnesses often experience abnormal oxygen supply and demand balances.^[1]^ Oxygen transport, oxygen utilization and oxygen metabolism disorders are the common pathophysiological basis of many critical cases. Potential and untreated overall organization and cell hypoxia is thought to donate to the growth of multiple organ failure or death. The detect as early as possible of global tissue hypoxia has significant consequences in the medical decision of critically ill patients. There may be some deficiencies in the commonly used indicators for the diagnosis of organ tissue hypoxia, like arterial oxygen saturation, venous oxygen saturation (S_v_O_2_), and blood lactate level. In the past, it was thought that the rapid increase of blood lactate level seriously ill was caused by the activation of anaerobic glycolysis pathway after insufficient tissue oxygen supply. According to previous research, hyperlactatemia considered as sign tissue hypoxia and hypoperfusion.^[2,3]^ Whereas blood lactate level is likely to rise through other mechanisms unrelated to tissue oxygen debt in critically ill.^[4,5]^Therefore, hyperlactatemia is easily disturbed by many factors when reflecting anaerobic metabolism secondary to systemic cellular and tissular hypoxia.

The hyperlactatemia and microcirculation disorders are widespread in seriously ill patients,^[3]^ particularly those with sepsis all have varying degrees of oxygen transport and oxygen utilization disorders. The ratio of the venous to arterial oxygen content (CO_2_) tension difference over the arterial to venous oxygen content difference (P_V-A_CO_2_/C_A-V_O_2_) may as the surrogate of the respiratory quotient, it has been suggested that this ratio can be used as a sign of tissue anaerobic metabolism with faster response than lactate and less false positives in critically ill patients.^[6,7]^ In intensive care unit (ICU), the usual feature of critically ill is the multiple organ dysfunction, severe hemodynamic changes, and impaired tissue oxygen utilization as hypovolemia, decreased vascular tone, microcirculation disorders. Studies^[8,9]^ have shown that the severity of microcirculation damage is the bad prognosis in septic shock. Although the existing oxygen metabolism indexes such as lactic acid, S_v_O_2_ and venous to arterial carbon dioxide differential pressure (P_v-a_CO_2_) can present microscopic oxygen metabolism disorders to a certain extent, but tissue hypoperfusion may still exist after the above indexes reach the standard.^[10]^ Over the past few years, the intervention of micro-circulation in the field of serious diseases has received extensive attention. Some clinical microcirculatory image capturing and analysis technology is also helping to visually observe the microcirculation status of patients.^[11]^ However, limited by regional and medical conditions, this microcirculation imaging technology may not be easy to popularize, especially the early microcirculation status of patients is indiscernibly in critical patients after admittance. Few studies have focused on early identify microcirculation disturbance by using P_V-A_CO_2_/C_A-V_O_2_, and thus further studies take into account the discrepancy of seriously ill.

Thus, we conducted retroactively cohort research that is based on a vast, publicly available database named medical information mart for intensive care IV (MIMIC-IV). Our main aim of studied correlation of P_V-A_CO_2_/C_A-V_O_2_, lactate acid level and hospital mortality in critical patients within 24 hours of admission. We hypothesized that the influence of P_V-A_CO_2_/C_A-V_O_2_ level in different subsets of patients on mortality rates might differ, and the sensitive P_V-A_CO_2_/C_A-V_O_2_ range might also vary across blood lactate level subsets in ICU.

## 2. Methods

### 2.1. Population and data extraction

#### 2.1.1. Data source.

The Massachusetts institute of technology developed an open access database named MIMIC-IV (2.2 version), which covered the medical records of 431,231 in-patients who accepted treatment at the Beth Israel Deaconess Medical Center between 2008 and 2019.^[12]^ Latter are one of the foremost educational medical services and referral center in the Boston, whereby seventy-seven critical care hospital beds are contained. Researchers can filter demographic features, vital, lab results, imaging tests of every single sick through unique identification granted during admission. Guo has finished its Collaborative Institutional Educational Initiative curriculum course (Certification number 52822183). Since the MIMIC-IV database is an open access available anonymized database, permission from the ethics committee was not necessary.

Study was that did not require our institutional review board endorsement because this research used one open access de-identified data from preexisting institutional review board endorsement. Our survey has been reported in line with the RE-porting of studies Conducted using Observational Routinely collected health Data,^[13]^ strengthening the reporting of observational studies in epidemiology declaration.^[14]^

#### 2.1.2. Patient population.

In our study, we extracted parameters for a number of patients from the database, including: basic demographic features (gender and age); blood PH, white-blood cell (WBC), blood anion-gap, blood creatinine, blood urea nitrogen, blood glucose, blood potassium, the sequential organ failure assessment (SOFA) score, the acute physiology score III, the logistic organ dysfunction system (LODS), the oxford acute severity of illness score (OASIS), the simplified acute physiology score II (SAPSII), system inflammatory reaction syndrome (SIRS) within the firstly twenty 4 hours after ICU admission; the anamnesis include chronic obstructive pulmonary disease (COPD), myocardial infarct, diabetes, coronary atherosclerotic heart disease, hypertension, congestive heart failure, peripheral vascular disease, liver disease, renal disease, cerebrovascular disease, and acute hepatic failure, acute kidney failure, acute respiratory failure; the P_V-A_CO_2_/C_A-V_O_2_ and lactate maximum within the first twenty 4 hours after admission in ICU; used of insulin, vasoactive agents drug, renal replacement therapy during hospitalization; occurrence of septic-shock or sepsis during hospitalization in ICU; mortality within 7 days, mortality within 28 days, ICU mortality of patient. The International Classification of Diseases Ninth Revision and Tenth Revision were used for disease diagnosis and identification.

**Figure 1.**
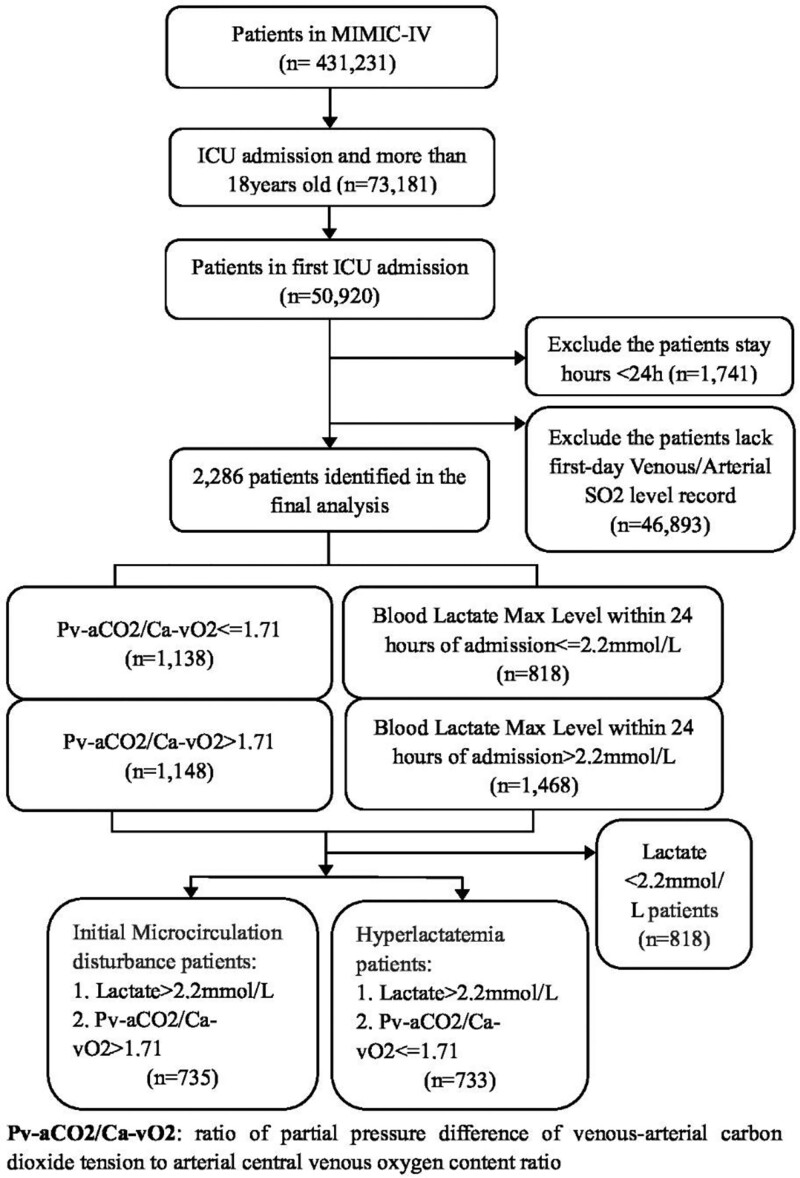
Flow chart of patient selection from medical information mart for intensive care IV (MIMIC-IV), Medical Information Mart for Intensive Care.

All adult seriously ill who eighteen years of age is or older in ICU have been gradual examined to be analyzed. We ruled out some patients who remained hours less than twenty-four in ICU. Patients were ruled out if they had disappeared first-day venous/arterial oxygen saturation records. The flowchart is shown in Figure 1.

### 2.2. Ratio of partial pressure difference of venous-arterial carbon dioxide tension to arterial venous oxygen content ratio definition

For each included patient, we gather the maximum lactate recorded by arterial blood gas within 24 hours of admission and stratified it as: hyperlactatemia (>2.2 mmol/L) and non-hyperlactatemia (≤2.2 mmol/L) based on previous studies.^[2,15]^ Meanwhile, the SvO_2_, carbon dioxide partial pressure (PvCO_2_) and hemoglobin collected at the same time on the day of admission were collected firstly, which was measured by the internal jugular catheter, and then the matching arterial oxygen saturation and carbon dioxide partial pressure were collected (PACO_2_) collected during the same time period, and finally the venous oxygen content and arterial oxygen content were calculated respectively, and the calculation formula according to the previous research method^[16]^ was as follows:

Oxygen content (CO_2_) = (Hb × SO_2_ × 1.36) + (PO_2_ × 0.0031)

According to the obtained oxygen content, P_V-A_CO_2_/C_A-V_O_2_ can be calculated with the following formula:

P_V-A_ CO_2_/C_A-V_ O_2_ = PVCO_2_ − P_A_CO_2_/C_A_O2 − C_V_O_2_

Due to an absence of universally acceptable medical criteria to grading the ratio of the venous-to-arterial CO2 tension difference over the arterial-to-venous oxygen content difference (P_V-A_ CO_2_/C_A-V_ O_2_) levels of seriously ill patients, we clustered P_V-A_ CO_2_/C_A-V_ O_2_ into 2 categories according to the median level (low: ≤1.71; high: >1.71).

### 2.3. Statistical analysis

Current retrospective analysis of gathered experimental dataset was layered pursuant to P_V-A_ CO_2_/C_A-V_ O_2_ and Lactate Max Level. Be collected data was performed by the normality test (Agostino tests), followed by describing analyzation of it. Uninterrupted data displayed as in mean (standard deviation) while non-parametric data were displayed as median (interquartile ranges, IQR) and were analyzed using the one-way ANOVA examination or non-parametric Kruskal-Wallis assay. Categorical data were cast as a frequency (percentage) and were compared using the X^2^ or rank-sum tests. All the variables included in this study had missing values of <1%, and these missing values will be replaced by modes. Atypical data were identified as values larger than the 99th or lower than the first percentile. Variables with outliers were winsorized utilizing Winsor2 command at STATA programs.

We conducted survival analyzation that use Cox Proportional-Hazards models with 3 kinds different confounder adjustment methods. Model 1 set nothing potential confounders. Model 2 corrected age and gender variable. Model 3 adjusted potential variables such as age, gender, SOFA score, peak number of WBC, maximum number of anion gap, maximum of creatinine. Hazards ratio (HR) and 95% confidence intervals was informed in Cox regression analysis. We also held sensitivity analysis for 7-day mortality outcome and 28-day mortality.

We produced Kaplan–Meier curves to show odds of survival around initial microcirculation disturbance and hyperlactatemia patient group. Entire statistical assessments were done by using STATA 17.0 (College Station, Texas) software. The *P* values with < .05 was taken as statistical meaning.

## 3. Results

### 3.1. Cohort characteristic

In MIMIC-IV, in total of 2.286 patients fulfilled the inclusion criteria. Of the study cohort, 1.468 patients had a blood lactate level more than 2.2 mmol/L on the first day and identified as hyperlactatemia, and the remaining 818 patients blood lactate level ≤2.2 mmol/L. Meanwhile, 1148 of these patients P_V-A_ CO_2_/C_A-V_O_2_ >1.71, and other 1138 not >1.71.

Table 1 showed the baseline characteristics for P_V-A_ CO_2_/C_A-V_ O_2_ and lactate levels groups, respectively. On the one hand, patients with hyperlactatemia within 24 hours of admission more critically ill than those with a lactate of <2.2 mmol/L, such as the statistically significant differences in curing, laboratory, severe scores, outcomes, and some diseases (COPD, liver disease, congestive heart failure, acute kidney failure). On the other hand, patients with lower P_V-A_ CO_2_/C_A-V_ O_2_ are older and have higher rates of cardiovascular system-related diseases and acute respiratory failure, but patients with higher P_V-A_ CO_2_/C_A-V_ O_2_ have higher SOFA, acute physiology score III, and OASIS score indices and are more likely to receive renal replacement therapy and insulin therapy. More importantly, patients in the high P_V-A_ CO_2_/C_A-V_ O_2_ group had higher morbidity of sepsis, septic shock, and in-hospital mortality. There were no clear differences in gender, some diseases (diabetes mellitus, COPD, liver disease, kidney disease, cerebrovascular disease, acute liver failure, acute renal failure), laboratory, vasopressor drug use, and some severe scores (SOFA, LODS, SAPSII, OASIS, SIRS) between the 2 groups with high and low P_V-A_ CO_2_/C_A-V_ O_2_.

**Table 1:** Baseline demographic and clinical characteristics by P_v-a_CO_2_/C_a-v_O_2_ and lactate max level in intensive care unit (ICU) patients.

Features	Ratio of partial pressure difference of venous-arterial carbon dioxide tension to arterial venous oxygen content ratio		*P* value	Blood Lactate Max Level within 24 h of admission		*P* value
	P_v-a_CO_2_/C_a-v_O_2_ ≤1.71	P_v-a_CO_2_/C_a-v_O_2_ > 1.71		Lactate ≤2.2 mmol/L	Lactate >2.2 mmol/L	
	n = 1,138	n = 1,148		n = 818	n = 1,468	
Demographic features						
Age (yr)	68.54 (59.17, 77.82)	66.74 (56.43, 77.33)	.017	67.73 (57.69, 76.75)	67.85 (57.35, 77.80)	.986
Gander			.897			.136
Female	451 (39.63%)	458 (39.89%)		342 (41.80%)	567 (38.62%)	
Male	687 (60.36%)	690 (60.10%)		476 (58.19%)	901 (61.37%)	
Comorbidity						
Diabetes	386 (33.91%)	373 (32.49%)	.469	269 (32.88%)	490 (33.37%)	.810
COPD	336 (29.52%)	356 (30.10%)	.440	305 (37.28%)	387 (26.36%)	<.001
CAHD	523 (45.95%)	405 (35.27%)	<.001	322 (53.13%)	606 (41.28%)	.371
Hypertension	326 (28.64%)	281 (24.47%)	.024	212 (25.91%)	395 (23.84%)	.607
Liver disease	241 (21.17%)	259 (22.56%)	.424	116 (14.18%)	384 (26.15%)	<.001
Renal disease	305 (26.80%)	306 (26.65%)	.937	232 (28.36%)	379 (25.81%)	.188
Cerebrovascular disease	142 (12.47%)	115 (10.01%)	.063	99 (27.62%)	158 (10.76%)	.331
Myocardial infarct	329 (28.91%)	308 (26.82%)	.267	211 (25.79%)	426 (29.01%)	.099
Congestive heart failure	546 (47.97%)	476 (41.46%)	.002	388 (47.43%)	634 (43.18%)	.050
Acute hepatic failure	147 (12.91%)	167 (14.54%)	.258	52 (6.35%)	262 (17.84%)	<.001
Acute kidney failure	616 (54.13%)	639 (55.05%)	.462	414 (50.61%)	847 (57.69%)	.002
Acute respiratory failure	470 (41.30%)	560 (48.78%)	<.001	385 (47.06%)	645 (43.93%)	.150
Curing						
RRT	233 (20.47%)	284 (24.73%)	.015	139 (16.99%)	378 (25.74%)	<.001
Insulin	949 (83.39%)	916 (70.79%)	.026	622 (76.03%)	1243 (84.67%)	<.001
Vasoactive drug	740 (65.02%)	744 (64.80%)	.913	419 (51.22%)	1065 (72.54%)	<.001
Laboratory*						
PH	7.37 (7.31, 7.42)	7.35 (7.27, 7.41)	<.001	7.37 (7.31, 7.42)	7.36 (7.28, 7.41)	<.001
WBC_max (×10^3^/uL)	15.20 (11.00, 20.60)	15.00 (10.40, 20.25)	.116	13.00 (9.30, 17.40)	16.50 (11.90, 22.40)	<.001
BUN_max (mg/dL)	25.00 (17.00, 42.00)	28.00 (17.00, 45.00)	.058	27.00 (18.00, 44.00)	22.00 (16.00, 39.00)	<.001
Anion Gap_max (mEq/L)	16.00 (13.00, 20.00)	17.00 (14.00, 21.00)	.003	15.00 (13.00, 18.00)	18.00 (14.00, 22.00)	<.001
Creatinine_max (mg/dL)	1.30 (0.90, 2.10)	1.45 (0.90, 2.40)	.049	1.20 (0.90, 2.10)	1.50 (1.00, 2.30)	<.001
Glucose_max (mg/dL)	154.00 (123.00,209.00)	157.00 (124.00,221.00)	.197	143.00 (118.00,190.00)	165.00 (127.00,239.00)	<.001
Potassium_max (mEq/L)	4.60 (4.20, 5.10)	4.60 (4.20, 5.20)	.628	4.50 (4.10, 5.00)	4.70 (4.30, 5.30)	<.001
Severe scoring						
SOFA	8.00 (5.00, 10.00)	8.00 (5.00, 11.00)	.056	6.00 (4.00, 8.00)	9.00 (6.00, 12.00)	<.001
APSIII	54.00 (38.00, 72.00)	56.00 (40.00, 75.00)	.026	49.00 (36.00, 63.00)	59.00 (41.00, 80.00)	<.001
LODS	6.00 (5.00, 9.00)	7.00 (5.00, 9.00)	.415	6.00 (4.00, 8.00)	7.00 (5.00, 10.00)	<.001
SAPSII	44.00 (35.00, 56.00)	45.00 (36.00, 57.00)	.147	41.00 (32.00, 51.00)	48.00 (38.00, 59.00)	<.001
OASIS	35.00 (29.00, 41.00)	36.00 (30.00, 42.00)	.053	34.00 (28.00, 40.00)	36.00 (31.00, 44.00)	<.001
SIRS	3.00 (2.00, 4.00)	3.00 (2.00, 4.00)	.976	3.00 (2.00, 3.00)	3.00 (3.00, 4.00)	<.001
Outcomes						
Sepsis	382 (33.56%)	450 (39.19%)	.005	284 (34.71%)	548 (37.32%)	.214
Septic Shock	302 (26.53%)	375 (32.66%)	.001	208 (25.42%)	469 (31.94%)	.001
Mortality 7 d	110 (9.66%)	141 (12.28%)	.045	39 (4.76%)	212 (14.44%)	<.001
Mortality 28 d	272 (23.90%)	332 (28.91%)	.007	159 (19.43%)	445 (30.31%)	<.001
Mortality hospital	290 (25.48%)	336 (29.26%)	.042	168 (20.53%)	458 (31.19%)	<.001

APSIII = Acute Physiology Score III, BUN = Blood Urea Nitrogen, CAHD = coronary atherosclerotic heart disease, COPD = chronic obstructive pulmonary disease, LODS = logistic organ dysfunction system, OASIS = oxford acute severity of illness score, RRT = renal replacement therapy, SAPSII = Simplified Acute Physiology Score II, SIRS = System Inflammatory Reaction Syndrome, SOFA = Sequential Organ Failure Assessment, WBC = white blood cell.

Vasoactive drug: norepinephrine, epinephrine, phenylephrine, vasopressin, and dopamine dobutamine or milrinone.

* Laboratory use the maximum value in the first 24 h after admission.

Table 2 is that we divided patients into the initial microcirculation disturbance group and the group based on Table 1. It is clearly that patients in the initial microcirculation disturbance group are younger and more likely to have acute respiratory failure, and the laboratory has higher values in addition to WBC and glucose max, as well as higher SOFA scores and sepsis, sepsis shock, in-hospital mortality. Was no difference between the remaining factors between 2 groups.

**Table 2:** Association of outcomes in patients with microcirculation disturbance and hyperlactatemia.

Variables	Initial microcirculation disturbance	Hyperlactatemia	*P*
	n = 735	n = 733	
Age (yr)	66.16 (56.08, 77.21)	68.97 (59.38, 78.76)	.002
Male	454 (61.76%)	447 (60.98%)	.757
Myocardial infarct	206 (28.02%)	220 (30.01%)	.402
Congestive heart failure	144 (19.59%)	118 (16.09%)	.080
Acute hepatic failure	147 (20.00%)	167 (22.78%)	.258
Acute kidney failure	425 (57.82%)	416 (56.75%)	.679
Acute respiratory failure	349 (47.48%)	296 (40.38%)	.006
RRT	205 (27.89%)	173 (23.60%)	.060
PH	7.35 (7.26, 7.41)	7.37 (7.31, 7.47)	<.001
WBC_max (×10^3^/uL)	16.50 (11.60, 22.50)	16.50 (12.10, 22.40)	.595
BUN_max (mg/dL)	29.00 (18.00, 44.00)	25.00 (17.00, 41.00)	.015
Anion Gap_max (mEq/L)	18.00 (15.00, 23.00)	17.00 (14.00, 22.00)	.004
Creatinine_max (mg/dL)	1.60 (1.00, 2.60)	1.40 (1.00, 2.20)	.004
Glucose_max (mg/dL)	170.00 (130.00, 243.00)	162.00 (126.00, 230.00)	.057
Severe scoring			
SOFA	10.00 (6.00, 12.00)	9.00 (6.00, 11.00)	.003
APSIII	61.00 (42.00, 82.00)	57.00 (41.00, 79.00)	.063
LODS	8.00 (5.00, 10.00)	7.00 (5.00, 10.00)	.304
SAPSII	48.00 (38.00, 60.00)	47.00 (38.00, 59.00)	.366
OASIS	36.00 (31.00, 45.00)	36.00 (30.00, 43.00)	.474
SIRS	3.00 (3.00, 4.00)	3.00 (2.00, 4.00)	.589
Outcomes			
Sepsis	293 (39.86%)	255 (34.78%)	.044
Septic shock	256 (34.82%)	213 (29.05%)	.018
Mortality 7 d	119 (16.19%)	93 (12.68%)	.056
Mortality 28 d	245 (33.33%)	200 (27.28%)	.012
Mortality hospital	244 (33.19%)	214 (29.19%)	.098

APSIII = acute physiology score III, BUN = blood urea nitrogen, LODS = logistic organ dysfunction system, OASIS = oxford acute severity of illness score, RRT = renal replacement therapy, SAPSII = simplified acute physiology score II, SIRS = system inflammatory reaction syndrome, SOFA = sequential organ failure assessment, WBC **=** white blood cell.

### 3.2. Mortality outcome and survival analysis

**Figure 2.**
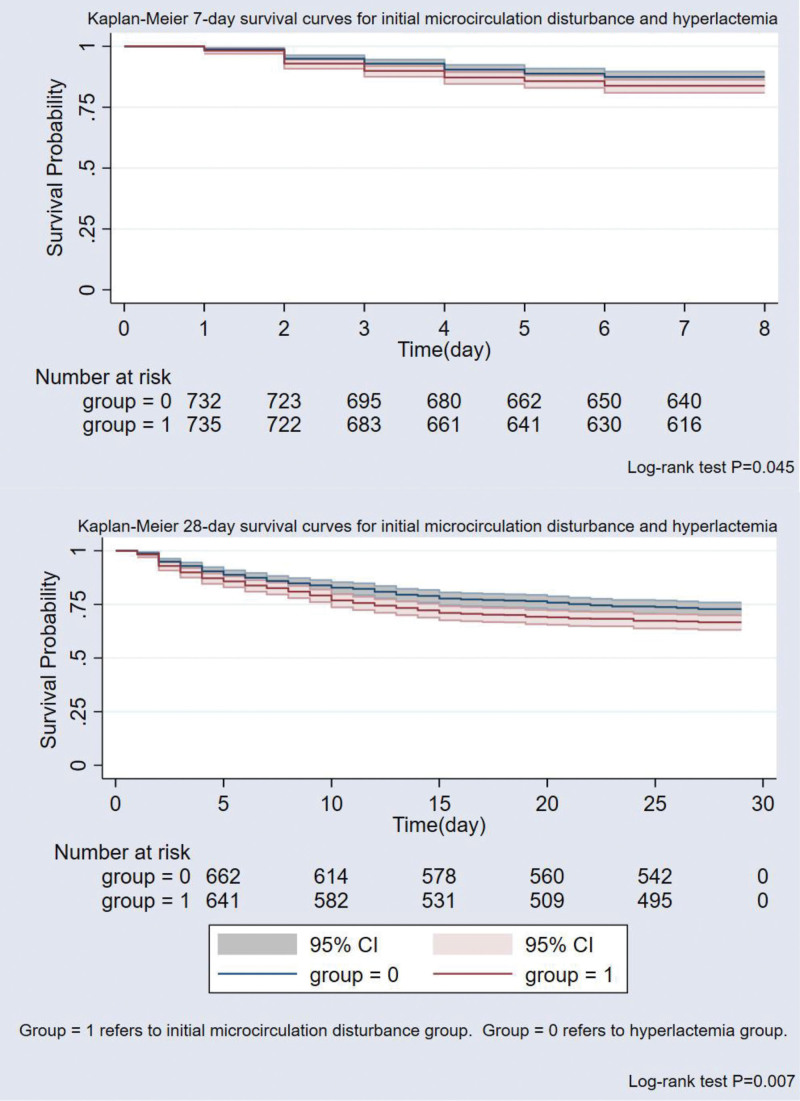
Kaplan–Meier survival estimates.

The results revealed that initial microcirculation disturbance patients was higher probability occurred 28-day mortality compare with hyperlactatemia in blood lactate high levels (>2.2 mmol/L) population (33.33% vs 27.28%; adjusted HR 1.34, 95% CI 1.11–1.61, *P* = .002 in model 2 & adjusted HR 1.24, 95% CI 1.02–1.50, *P* = .026 in model 3), and the initial microcirculation disturbance patients compare with hyperlactatemia patients in 7-day mortality is more (16.19% vs 12.68%; adjusted HR 1.35, 95% CI 1.03–1.78, *P* = .029 in model 2 & adjusted HR 1.23, 95% CI 0.93–1.63, *P* = .139 in model 3). Similarly, sepsis and septic shock have a higher incidence in initial microcirculation disturbance populations (Table 2). Kaplan–Meier curves of 7 (28)-day survival were displayed in Figure 2. We noted initial microcirculation disturbance was the more increased risk of mortality rate in Cox regression analysis especially in model 2.

In following sensitivity research, we separately assessed the relationship between the initial microcirculation disturbance group with hyperlactatemia group and 7 (28)-day mortality with Cox Proportional-Hazards models (Table 3). In basic study, initial microcirculation disturbance group had a 31% and 28% increase in 7 (28)-day mortality. Interestingly, after adjusting the possible confounding, the in the original microcirculation disturbance group showed a lower increase in the 7 (28)-day mortality risk (HR 1.23, 95% CI 0.93–1.63, *P* = .139 & HR 1.24, 95% CI 1.02–1.50, *P* = .026), and the mortality risk fell 4% compare with crude model. But, when we only adjusting for age and gender confounders, the in initial microcirculation disturbance group showed substantial increase in 7 (28)-day mortality risk (HR 1.35, 95% CI 1.03–1.78, *P* = .002 & HR 1.34, 95% CI 1.11–1.61, *P* = .002).

**Table 3:** Association between whether there are initial microcirculatory disorders conditions and 7 (28)-d mortality by Cox regression model.

	N	Model 1	*P*	Model 2	*P*	Model 3	*P*
		HR (95%Cl)		HR (95%Cl)		HR (95%Cl)	
7-d mortality							
Hyperlactatemia	733	0.76 (0.58–0.99)	.050	0.73 (0.56–0.96)	.029	0.80 (0.61–1.07)	.139
Initial microcirculation disturbance	735	1.31 (1.00–1.72)	.050	1.35 (1.03–1.78)	.029	1.23 (0.93–1.63)	.139
28-d mortality							
Hyperlactatemia	733	0.77 (0.67–0.93)	.009	0.74 (0.61–0.89)	.002	0.80 (0.66–0.97)	.026
Initial microcirculation disturbance	735	1.28 (1.06–1.54)	.009	1.34 (1.11–1.61)	.002	1.24 (1.02–1.50)	.026

Model 1 not adjusted.

Model 2 adjusted for age, gender.

Model 3 adjusted for age, gender, SOFA score, maximum number of WBC within 24 h of admission, maximum number of anion gap within 24 h of admission, maximum number of creatinine within 24 h of admission.

CI = confidence interval, HR = hazards ratio.

### 3.3. Secondary outcomes

The comparing of secondly outcomes between the groups are displayed in Table 2. Initial microcirculation disturbance patients have a higher probability of developing sepsis and septic shock during hospitalization than hyperlactatemia patients (39.86% vs 34.78%, *P* = .044 & 39.82% vs 29.05%, *P* = .018). At the same time, we noticed that the initial microcirculation disorder group had higher SOFA and acute physiology score III scores than the hyperlactatemia group alone, however, there was no significant difference in LODS, SAPSII, OASIS and SIRS scores.

## 4. Discussion

Our study indicated that initial microcirculation disturbance status was linked to a higher death rate in overall critical condition demographic, especially with a rise in 28-day mortality. The initial microcirculation disturbance status had increased risk of sepsis and septic shock during hospitalization.

Although lactic acid has been used for decades to anticipate the prognosis of severely ill,^[17,18]^ but due to the many influencing factors of lactic acid^[19]^ and the results of the research are controversial. So, we added the P_V-A_ CO_2_/C_A-V_ O_2_ to the lactic acid value to investigate whether initial microcirculation disturbance status is linked to poor prognosis in severely ill demographic. In presently, just few studies were readily available concentrated on the utilization of initial microcirculation disturbance status in critically ill patients. Our research comes as an integrated reassessment of the initial microcirculation disturbance for critically ill patients by using a wide-open database, which supplied widespread and authenticity for the initial microcirculation disturbance of chosen demographic. Our results suggested that had initial microcirculation disturbance status was associated with unfavorable results seriously ill patients.

On reviewing previous studies, an observational study published in 2004 by Yasser et al found that serum lactate can be used as an indicator of the perfusion of the microcirculation in critically illness, and lactate is associated with microcirculation status and mortality in patients with septic shock.^[20]^ And, the microcirculation is governed by complex interaction of many neuroendocrinological, paracrine, and mechanosensory pathways, which also include disturbances in capillary flow and changes in perfusion vessel density.^[9]^ However a retrospective study conducted in 2016, Jean-Louis et al reported that blood lactate level, although a good prognostic marker, changed relatively slowly over time and was susceptible to the influence of underlying diseases.^[21]^ In the same year, Shin et al shown a single-center retrospective research and found that the complementation of initial lactate level and SvO_2_ level in critical condition could be used as a correlation index of comprehensive evaluation of body oxygenation status and 28-day mortality in patients with severe sepsis or septic shock.^[22]^ Recently retrospective observational research by Gao et al revealed that by Cox regression analysis of the 28-day mortality showed that P_V-A_ CO_2_/C_A-V_ O_2_ ratio (Relative risk (RR): 3.888, 95% CI: 2.443–6.189, *P* < .001) and LCR (RR: 0.073, 95% CI: 0.008 − 0.640, *P* = .018) was independent predictors of mortality at day 28.^[23]^ Our study also showed that initial microcirculation disturbance status with increased risk of 7 (28)-day mortality in critically ill patients (HR 1.35, 95% CI 1.03–1.78, *P* = .029 & HR 1.34, 95% CI 1.11–1.61, *P* = .002), which backed a finding of Gao et al with a bigger sample from MIMIC-IV database.

And the critically demographic, especially in pathophysiological conditions where shock may occur, enhanced tissue anaerobic metabolism leads to increased CO_2_ release and increased P_V-A_ CO_2_. Because C_A-V_ O_2_ can be used as a substitute for tissue oxygen uptake, considering that oxygen molecules in critically ill patients cannot form a gradient difference in oxygen concentration to enter cells through the blood in the form of diffusion under normal conditions, when tissue edema, the diffusion spacing increases, and the cells appear hypoxia,^[24]^ resulting in a decrease in C_A-V_ O_2_. So, the increase of P_V-A_ CO_2_/C_A-V_ O_2_ may indirectly indicate that the degree of anaerobic metabolism of patients is enhanced, and the body is in a state of relative hypoxia. Moreover, the further correction of P_V-A_ CO_2_ by C_A-V_ O_2_ can further increase the degree of anaerobic metabolism. It is interesting that many scholars have different judgment of the critical value of P_V-A_ CO_2_ by C_A-V_ O_2_ ratio in different clinical studies,^[7,25–27]^ which may be due to more variables in the formula resulting in increased measurement error and uncertainty of the results.

In our retrospective analysis, we first compared the link between maximum lactate level and the P_V-A_ CO_2_/C_A-V_ O_2_ ratio within 24 hours of admission to different outcomes in critically ill patients. Maximum lactate level was indeed a good predictor of poor outcomes. The P_V-A_ CO_2_/C_A-V_ O_2_ ratio also had predictive power for adverse outcomes. However, considering that there are many interfering factors of lactic acid, we further used the P_V-A_ CO_2_/C_A-V_ O_2_ ratio to determine whether there are early potential microcirculation disorders based on hyperlactacidemia. We observed the relationship between early potential microcirculation disorders and (7)28-day mortality of patients with critically ill by using univariate and multivariate analyses, in which early potential microcirculation disorders was used as an exposure variable and (7)28-day mortality as an outcome variable with other variables being adjusted. Then, we discovered that patients with premature underlying microcirculation had a higher (7)28-day mortality than those with isolated hyperlactatemia. This study had multiple strong points. For starters, this research included a significant demographic size in the real-world electronic medical records, which has offered generalizability and effectiveness for intensive care practice. For a further, research concentrated on early potential microcirculation disturbance patients bring innovative ideas and chances for septic shock of critically ill. Microcirculation disturbance was associated with increasing risk of mortality, stratifying patients immediately based upon the disturbance condition after urgent admission did matter.

And in the meantime, there are certain limitations should be considered in the analysis: Since this is clinical observational retrospective research, some unmeasured covariates may have residual confounding and may be affected to varying degrees by these factors. Given the nature of an observational study, we can just examine connections and explain relationships between detectable factors, rather than unmeasurable confounders, so broader, more evidence-based clinical studies in larger populations are necessary to confirm our conclusions. The study did not investigate the lack of ethnicity, so researchers must be cautious when inferring our conclusions to individual populations. As this study is ancillary data analysis based upon a huge, multicentre intensive-care database, we cannot contain patients in the actual clinical scenario. The study is preliminary relation between early microcirculatory disorders and mortality, but some factors change dynamically over time, and more accurate stratification and dynamic analysis have better accurate predictions. Due to the limitations of the database itself, the venous blood we used cannot distinguish between superior or inferior vena cava and may have missed the assessment of some important visceral oxygenation.

## 5. Conclusion

Our study suggests that patients with early underlying microcirculatory disturbances have a higher risk of adverse outcomes than those with early hyperlactatemia alone. In other words, the combination of P_V-A_ CO_2_/C_A-V_ O_2_ ratio and blood lactate is a better predictor of the risk of adverse outcomes in critically ill patients than lactate level alone.

## Author contributions

**Conceptualization:** Guo Tongwu, Yuanzheng Yang.

**Data curation:** Guo Tongwu.

**Formal analysis:** Guo Tongwu.

**Funding acquisition:** Huanying Yi.

**Methodology:** Guo Tongwu.

**Project administration:** Yuanzheng Yang.

**Software:** Guo Tongwu.

**Supervision:** Yuanzheng Yang.

**Validation:** Rui Zheng.

**Writing – original draft:** Guo Tongwu.Writing – review & editing: Rui Zheng, Yuanzheng Yang.
